# BiNoM 2.0, a Cytoscape plugin for accessing and analyzing pathways using standard systems biology formats

**DOI:** 10.1186/1752-0509-7-18

**Published:** 2013-03-01

**Authors:** Eric Bonnet, Laurence Calzone, Daniel Rovera, Gautier Stoll, Emmanuel Barillot, Andrei Zinovyev

**Affiliations:** 1 Institut Curie, 26 rue d’Ulm, Paris, F-75248, France; 2 INSERM, U900, Paris, F-75248, France; 3 Mines ParisTech, Fontainebleau, F-77300, France

**Keywords:** Systems biology, Cytoscape, Software, SBML, BioPAX, CellDesigner, Conversion, SBGN, Reactome, Network analysis, Path analysis, Molecular maps, Pathways

## Abstract

**Background:**

Public repositories of biological pathways and networks have greatly expanded in recent years. Such databases contain many pathways that facilitate the analysis of high-throughput experimental work and the formulation of new biological hypotheses to be tested, a fundamental principle of the systems biology approach. However, large-scale molecular maps are not always easy to mine and interpret.

**Results:**

We have developed BiNoM (Biological Network Manager), a Cytoscape plugin, which provides functions for the import-export of some standard systems biology file formats (import from CellDesigner, BioPAX Level 3 and CSML; export to SBML, CellDesigner and BioPAX Level 3), and a set of algorithms to analyze and reduce the complexity of biological networks. BiNoM can be used to import and analyze files created with the CellDesigner software. BiNoM provides a set of functions allowing to import BioPAX files, but also to search and edit their content. As such, BiNoM is able to efficiently manage large BioPAX files such as whole pathway databases (e.g. Reactome). BiNoM also implements a collection of powerful graph-based functions and algorithms such as path analysis, decomposition by involvement of an entity or cyclic decomposition, subnetworks clustering and decomposition of a large network in modules.

**Conclusions:**

Here, we provide an in-depth overview of the BiNoM functions, and we also detail novel aspects such as the support of the BioPAX Level 3 format and the implementation of a new algorithm for the quantification of pathways for influence networks. At last, we illustrate some of the BiNoM functions on a detailed biological case study of a network representing the G1/S transition of the cell cycle, a crucial cellular process disturbed in most human tumors.

## Background

Biological pathways and networks comprise sets of interactions, or functional relationships, occurring at the molecular level in living cells [1,2]. A large body of knowledge on cellular biochemistry is organized in publicly available repositories such as the KEGG database [3], Reactome [4], MINT [5], or the Cancer Cell Map (http://cancer.cellmap.org/). All these pathway and biological network databases facilitate a large spectrum of analyses, improving our understanding of cellular systems. For example, it is now a very common practice to cross the output of high-throughput experiments, such as mRNA or protein expression levels, with curated biological pathways in order to visualize changes, analyze their impact on a network and formulate new hypotheses about biological processes [6,7]. The development of those pathway repositories has also fueled the creation of standard representations and formats, to facilitate the exchange and representation of data, such as the Biological Pathway Exchange standard (BioPAX) [8], the Systems Biology Markup Language (SBML) [9] or the Systems Biology Graphical Notation (SBGN) [10]. The Pathguide website counts more than 300 web-accessible biological pathway and network databases [11], many of which are using the SBML and BioPAX standard formats. Ultimately, those integrated resources will facilitate computational model building, their exchange, re-usability and their experimental validation, a cycle that is the cornerstone of the systems biology approach [12-14].

As a consequence, there is a need for the precise and accurate construction of pathways and large-scale molecular maps covering fundamental biological processes. Such maps are often constructed by manual curation of the literature or automated curation from pathway databases [15]. More and more, they are focused on the regulation of biological processes involved in diseases such as cancer, Alzheimer’s disease or Crohn’s disease, to name a few [16-19]. However, the scale of such maps, even when they are focusing on a particular process, is quite large, with hundreds of chemical species and interactions. The analysis and interpretation of such maps is therefore not a straightforward task. Several computational tools have been developed to facilitate the visualization, curation and analysis of pathways [1]. For example, CellDesigner is a software package for the graphical editing of biological pathway diagrams [20]. CellDesigner files are using the SBML format specification, with specific extensions describing biological types of chemical species and the layout of the reaction graph. There is obviously a need for user-friendly software tools that would allow the user to easily import data from various standard format sources, to perform structural analyses on these pathways and to manipulate networks, and to be able to export a network to a suitable format for further analysis. We have created BiNoM [21], a software plugin for the popular Cytoscape network vizualization and analysis tool [22] precisely to fulfill this purpose. There are several tools available for the import, visualisation and export of standard systems biology file formats, as well as their their conversion [20,23-26]. There is also a significant number of tools for network analysis [27-31]. However, we think that the strength of BiNoM is to provide at the same time a strong support for a choice of systems biology file formats, a set of robust and powerful network analysis tools, and also some very speficic functions that are not available in any other tool at the moment (see Table 1 for a detailed comparison of BiNoM’s function with other tools). BiNoM is designed to be useful in a finite set of pragmatic, user-oriented and proved to be needed scenarios for biological networks analysis. For instance, a user may want to import a molecular map from a CellDesigner file, analyze it using graph-based algorithms, and finally export a subnetwork of interest to the SBML format for mathematical modeling using a dedicated software. Obviously, it is rather complicated to provide robust support for all the standard systems biology formats that are now available. We have therefore implemented functions in BiNoM for importing and exporting from and to a selection of file formats (CellDesigner, BioPAX Level 3, CSML, SBML, see Table 2 for a detailed information on the exact import/export possibilities of BiNoM). BiNoM uses its own ontology for the graphical representation of the different entities and their relationships. The graphical conventions in BiNoM are inspired by the ones defined for the SBGN standard (see the BiNoM manual chapter 8 for a more complete description). BiNoM also implements several functions based on graph operations for the structural analysis of biological networks. Those functions can be used to reduce the complexity and extract meaningful subnetworks from large-scale molecular maps. Here, we provide a detailed view on the functions implemented in BiNoM that permit specific extraction of information from molecular maps and improve their readability and usability. We also highlight novel functions that were implemented recently, such as the support of the latest BioPAX specification (BioPAX Level 3) and an algorithmic approach for the quantification of pathways on influence networks (PIQuant, Pathway Influence Quantification algorithm). We illustrate the use of the principal BiNoM functions with a detailed analysis of a molecular network of the G1/S transition of the cell cycle, a central mechanism for tumor development and progression.

**Table 1:** Comparison of BiNoM functionalities with other software tools

**Function**	**BiNoM**	**CellDesigner**	**BioPAX2SBML**	**SyBiL**	**SBFC**	**Biographer**	**CySBML**	**ShortestPath**	**Glay**	**MCode**	**Moduland**	**ClusterMaker**	**NeMo**
Standalone application	∘	•	•	•	•	•	∘	∘	∘	∘	∘	∘	∘
Cytoscape plugin	•	∘	∘	∘	∘	∘	•	•	•	•	•	•	•
BioPAX import	•	•	•	•	•	∘	∘	∘	∘	∘	∘	∘	∘
BioPAX visualization	•	•	∘	•	∘	∘	∘	∘	∘	∘	∘	∘	∘
BioPAX properties editor	•	∘	∘	∘	∘	∘	∘	∘	∘	∘	∘	∘	∘
BioPAX queries	•	∘	∘	∘	∘	∘	∘	∘	∘	∘	∘	∘	∘
BioPAX export	•	∘	∘	•	•	∘	∘	∘	∘	∘	∘	∘	∘
SBML import	•	•	∘	•	•	•	•	∘	∘	∘	∘	∘	∘
SBML visualization	•	•	∘	•	∘	•	•	∘	∘	∘	∘	∘	∘
SBML validation	∘	∘	∘	∘	•	∘	•	∘	∘	∘	∘	∘	∘
SBML layout and													
qualitative support	∘	∘	•	∘	∘	∘	•	∘	∘	∘	∘	∘	∘
CellDesigner import,													
visualization and export	•	•	∘	∘	∘	∘	∘	∘	∘	∘	∘	∘	∘
CSML import	•	∘	∘	∘	∘	∘	∘	∘	∘	∘	∘	∘	∘
CSML visualization	•	∘	∘	∘	∘	∘	∘	∘	∘	∘	∘	∘	∘
Cytoscape extra													
utilities	•	∘	∘	∘	∘	∘	∘	∘	∘	∘	∘	∘	∘
Get connected													
network components	•	∘	∘	∘	∘	∘	∘	∘	∘	•	∘	•	•
Semantic clustering	•	∘	∘	∘	∘	∘	∘	∘	∘	∘	∘	∘	∘
Community structure													
clustering	∘	∘	∘	∘	∘	∘	∘	∘	•	∘	∘	∘	∘
Hierarchical modules													
clustering	∘	∘	∘	∘	∘	∘	∘	∘	∘	∘	•	∘	∘
Multiple standard													
clustering methods	∘	∘	∘	∘	∘	∘	∘	∘	∘	∘	∘	•	∘
Network decomposition	•	∘	∘	∘	∘	∘	∘	∘	∘	∘	∘	∘	∘
Get shortest paths	•	∘	∘	∘	∘	∘	∘	•	∘	∘	∘	∘	∘
Get optimal and suboptimal													
shortest paths	•	∘	∘	∘	∘	∘	∘	∘	∘	∘	∘	∘	∘
Get all non													
self-intersecting paths	•	∘	∘	∘	∘	∘	∘	∘	∘	∘	∘	∘	∘
Path influence quantification	•	∘	∘	∘	∘	∘	∘	∘	∘	∘	∘	∘	∘
Module manager	•	∘	∘	∘	∘	∘	∘	∘	∘	∘	∘	∘	∘

The table is comparing the main functions in BiNoM with other visualization and network analysis tools. The symbol • indicates that the function is available within the tool, while ∘ indicates that the function is not available. The references for the software tools are: CellDesigner [20,23], BioPAX2SBML [24], SyBiL [25], SBFC (Systems Biology File Converter (http://www.ebi.ac.uk/compneur-srv/sbml/converters/SBMLtoBioPax.html), biographer (http://code.google.com/p/biographer/), cySBML [26], ShortestPath (http://csresources.sourceforge.net/ShortestPath/), Glay [27], MCode [28], ModuLand [29], clusterMaker [30], NeMo [31].

**Table 2:** Detailed import/export BiNoM capabilities for standard systems biology file formats

**Import from**	**Export (from / to)**
CellDesigner v3.x, 4.1, 4.2	BioPAX Level 3 / BioPAX Level 3
BioPAX Level 3	BioPAX Level 3 / SBML Level 2
SBML Level 2	CellDesigner v3.x, 4.1, 4.2 / CellDesigner v4.1
CSML v3.0	CellDesigner v3.x, 4.1, 4.2 / BioPAX Level 3
	CellDesigner v3.x, 4.1, 4.2 / SBML Level 2
	CSML v3.0 / SBML Level 2

The table is indicating the different standard systems biology file formats and versions that can be currently imported/exported in BiNoM, as well as what type of conversions are possible.

## Implementation

BiNoM is implemented in the Java^TM^ programming language, as a plugin for the network visualization and analysis software package Cytoscape [22]. Although the primary use of BiNoM is through the Cytoscape software, the underlying logic of most of the BiNoM functions is completely decoupled from the Cytoscape objects, allowing developers to also use BiNoM as an independent Java library [21]. The installation of BiNoM can be done through the Cytoscape plugin manager (menu “Plugins > Manage Plugins”, Section “Other”, then select the latest version of BiNoM). Alternatively, the user can also download the plugin together with a manual and the source code from the BiNoM website (http://binom.curie.fr/).

BiNoM manipulates the information contained in standard systems biology files by mapping it onto a labeled graph, called index. The index does not try to map the totality of all details but rather serves as a connection map for the objects contained in other ontologies. The index contains the minimum information needed to graphically represent objects and connections between them. BiNoM index is a light-weight construction which can be easily regenerated, does not duplicate the information in existing files and serves only to facilitate the visualization and to access existing systems biology files. Currently, BiNoM index is mostly developed to map BioPAX ontology files and CellDesigner object schema. More specifically, we use XmlBeans (http://xmlbeans.apache.org/) to create Java classes from the xml definition file in order to access all the elements contained in the CellDesigner, SBML and CSML files. BioPAX uses the web ontology language specification (OWL, (http://www.w3.org/2004/OWL/) to store data in XML-formatted files. In BiNoM, we use the Jastor and the Jena Java libraries (http://jastor.sourceforge.net/), (http://jena.sourceforge.net/) to automatically create Java classes from the BioPAX specifications, allowing a convenient access to the different data types encoded in the BioPAX files. More detailed informations about the index and the mapping are available in the BiNoM manual (http://binom.curie.fr).

The core functions of BiNoM can be grouped in five different topics: Input/Output, Structural Analysis, BioPAX utils & query, Module manager and Utilities.

### BiNoM input / output

BiNoM functions facilitate the import and export of some of the standard systems biology file formats, but BiNoM plugin is not designed to be a universal converter (for a complete list of the different import/export possibilities in BiNoM, see Table 2). For instance, BiNoM will be useful in the examples of conversion and analysis scenarios detailed below (non-exhaustive list): 

• Interconversion of CellDesigner files to BioPAX, and from a BioPAX reaction network to SBML Level 2.

• Import of a BioPAX file as a reaction network and/or a pathway structure and/or an interaction map, followed by the creation of a subnetwork saved as a new BioPAX file.

• Import of a BioPAX file, selection of a subnetwork of interest saved as a SBML file for the creation of a computational model using an appropriate software package, such as CellDesigner [20] or GINsim [32].

• Import a large CellDesigner map and export only a subnetwork as a new CellDesigner file.

The BioPAX community has recently made a major update of the BioPAX standard, producing a new specification known as BioPAX Level 3 (http://www.biopax.org/). This format supports metabolic pathways, signaling pathways (including states of molecules and generic molecules), gene regulatory networks, molecular interactions and genetic interactions. Due to major changes in the specification, the BioPAX Level 3 is not backward compatible with the Level 2 file format.

**Figure 1:**
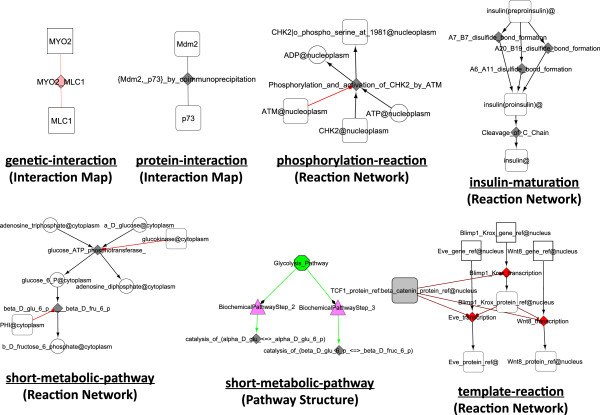
**Visualization of the six BioPAX example files, provided in BioPAX 3.0 documentation.** The BioPAX 3.0 documentation available at http://biopax.org contains six simple examples of BioPAX 3.0 files that describe different aspects of biological network interactions (genetic interaction, short metabolic pathway, gene regulatory network, biochemical reaction, phosphorylation, protein interaction). Here we show how BiNoM visualizes these examples after their import. The BiNoM type of representation is indicated below the reaction type, in brackets (Reaction Network, Pathway Structure and Interaction Map). The graphical node and edge semantic is described in more details in the BiNoM manual.

**Figure 2:**
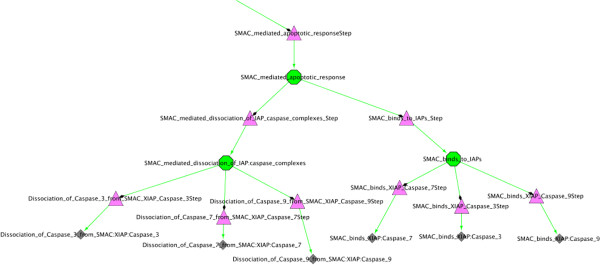
**Apoptosis pathway structure.** Zoom on a portion of the representation of BioPAX data extracted from the Reactome database [4], corresponding to the Apoptosis pathway and imported through BiNoM, using Pathway Structure BioPAX representation. The green nodes represent pathways, the pink triangular nodes denote steps, while grey nodes indicate reactions.

BioPAX Level 3 files are imported as three separate graphs, respectively the Reaction Network (RN), representing the biochemical reaction network, the Pathway Structure (PS), showing the hierarchical organisation of pathways, and the Interaction Map graph (IM). At the moment, the MIRIAM annotations are not imported in BiNoM, but we plan to provide access to this type information soon. Several examples of simple BioPAX Level 3 files imported through BiNoM, representing different types of interactions, are shown on Figure 1. Figure 2 shows the hierarchical structure of the human apoptosis pathway, extracted from Reactome database, and constructed by BiNoM.

When importing a file, BiNoM is calling a *naming service* function in order to create meaningful names for the various entities. More precisely, entity names are combined with other features such as modifications, compartment and complex components. The different features are indicated by special characters, such as “@” for the compartments, “ |” for modifications and “:” to delimitate the different members of a complex. For example, the name Cdc25 |Pho@cytoplasm represents the protein Cdc25 in a phosphorylated state, located in the cytoplasm, while the name Cdc13:Cdc2 |Thr167_pho@cytoplasm indicates a protein complex located in the cytosplasm, composed of the protein Cdc13 and the protein Cdc2 phosphorylated at position 167 on a threonine residue.

### BiNoM structural analysis

The central goal of the BiNoM plugin is to provide efficient methods and algorithms to reduce the inherent complexity of biological networks into manageable and meaningful subnetworks. This goal is achieved by a set of functions included as a built-in structural graph analysis library. Some of the functions take into account the semantics contained in the graph element names. The structural analysis functions implemented in BiNoM include the identification of connected and strongly connected components, pruning of the network, decomposition by involvement of a protein (material components) or by cyclic decomposition, path analysis and network clustering. We also introduce in this version of BiNoM a novel function to quantify the influence of a source node on a target node taking into account experimental data, called PIQuant. In the following paragraphs, we will detail network decomposition and the PIQuant score.

#### Decomposition by involvement of a protein or by cyclic decomposition

BiNoM proposes three methods to dissect a complex biological network into parts. A trivial approach to separate a network into subparts is to dissociate the unconnected subparts of the network. A more sophisticated one consists in decomposing the network into strongly connected components, using the algorithm of Tarjan [33]. It is also possible to prune the network into three different parts: the one with all the elements associated with the *input* part of the network (from which all paths lead to the central core), the second with all the elements associated with the *output* part (from which there are no paths leading back to the central core) and the last part with all the elements linked to the central core, the cyclic part, composed from strongly connected components, possibly connected together. This type of approach corresponds to finding the bow-tie graph structure [34].

**Figure 3:**
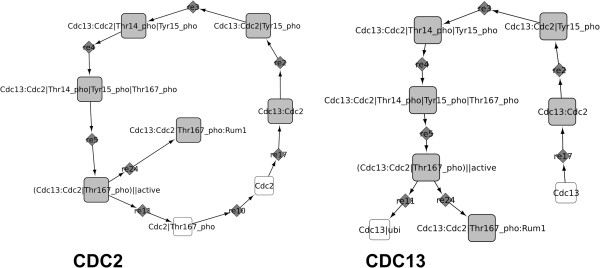
**Decomposition in material components.** The two overlapping subnetworks found after the decomposition in material components of the cell cycle model of Novak et al. [35], corresponding to the components Cdc13 and Cdc2.

The decomposition in material components is using the node name semantics to isolate subnetworks in which each protein is involved, either as a simple chemical species or as part of a complex. As a result, major overlaps between the different subnetworks are to be expected, as many proteins are expected to be involved in different complexes. Figure 3 shows two examples of subnetworks obtained by material component decomposition applied to a cell cycle network model of the yeast species *S. pombe*[35]. This approach identifies different parts of the life cycle of a given protein.

**Figure 4:**
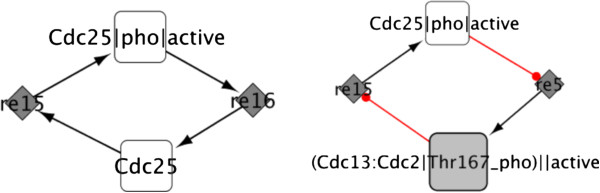
**Decomposition in cycles.** The figure shows two cycles for the CDC25 protein found after the decomposition of the cell cycle network model of Novak et al. [35].

The cycle decomposition is splitting the network into relevant directed cycles [36], using a modifed version of the algorithm of Vismara and colleagues [37]. This procedure commonly shows the different mechanisms in which the protein is playing a role. Care must be taken when applying this approach, as the number of cycles can be huge for large network structures. For example, it might be preferable to eliminate first the network hubs, which are by definition highly connected, and also group short cycles in larger subnetworks before applying the decomposition function. Figure 4 shows two cycles involving CDC25 after a cycle decomposition.

Obviously, the result of some decomposition functions will result in subnetworks that share some components, as it is for example often the case with the decomposition in material components. Therefore, BiNoM also includes a function to *cluster* networks, based on common components such as protein or protein complexes. To determine the size of the clusters, the user can specify a percentage of intersection (ranging from 0 to 100%) that will be used as a threshold to create the clusters.

#### Path analysis algorithms

BiNoM analysis functions also include classical path analysis algorithms, such as finding the shortest paths, the suboptimal shortest paths or all non self-intersecting paths (Table 3). The shortest path is calculated as the path having the minimal sum of weights of the edges composing the path (Dijkstra’s algorithm) while the suboptimal path is constructed by removing all edges of all shortest paths one by one, and finding the new shortest path. All non self-intersecting paths are those paths that do not contain loops (self-intersections). They are found using a variant of breadth-first search algorithm. The user should be careful when using this procedure, as the number of paths between nodes can be very large for big networks. In order to limit the number of paths found, BiNoM allows to specify the maximal length of the path to be found.

**Table 3:** BiNoM path analysis algorithms

**Algorithms**	**Directed**	**Finite**
	**paths search**	**radius**
Shortest paths	o	o
(Dijkstra’s algorithm)		
Optimal and suboptimal	o	o
shortest paths		
All non self-intersecting	o	o
paths		

Listing of the different algorithms implemented in BiNoM for path analysis. The “Directed paths search“ toggles the search for directed or undirected paths. The ”Finite radius” option lets the user restrict the search to a given path length, in order to limit the size of the results and computation time.

#### Pathway influence quantification algorithm

In this version of BiNoM, we have introduced a novel approach called PIQuant. It consists of associating a score to a target node of interest for a given network, that will quantify the effect of experimental data. A target node can be a gene, or a phenotype of interest, that represents a more complex biological function, such as cell proliferation or apoptosis. A positive or negative PIQuant score value is a quantitive theoretical prediction of the over or underexpression of the target node. For instance, let us consider that we have experimental data for a given network corresponding to differential gene expression values (e.g. disease/normal ratios). In that case, a positive or a negative PIQuant score for a given phenotype (target node) predicts quantitatively that the phenotype would be respectively enhanced or inhibited. Thus, the PIQuant score can be used to compare the effects of two different experimental datasets on the same phenotype (i.e. using the same network), or to compare the effects of two different network architectures on the same phenotype for one experimental dataset.

More formally, we define a node as *annotated* when a signed real number is assigned to the node, representing an experimental data value (e.g. the expression ratio of a gene between a disease and a normal state, obtained from transcriptomic profiling). A path *k* ∈ {1,…,*q*} is defined as the sequence of consecutive connected nodes between an annotated node and a target node (without repetition of any node or edge). We can extract a set of paths from annotated nodes to target nodes (indexed from 1 to *q*), by using various algorithms. In BiNoM, we propose three solutions to search for paths between the annotated and target nodes (shortest paths, suboptimal shortest paths and all non self-intersecting paths, see previous paragraph). The annotation *α*_*k*_ of the path *k* is defined as the annotation of the first node of the path. We define the sign *σ*_*k*_ of the path *k* as the product of the signs of every edge of the path and the length *λ*_*k*_ of the path *k* as the number of edges in the path. A summary of the input data types is shown in Table 4. We hypothesize that the longer the path is, the lesser the global influence will be on the target node. This assumption has the advantage of being simple and does not require the estimation or calculation of extra parameters. Considering a set of *q* paths that have been extracted from the network of interest, between a selection of annotated and target nodes defined by the user, the PIQuant score is then defined as:

**Table 4:** PIQuant score input data

**Data type**	**Description**
Influence network (set of paths)	An influence network of interest composed of different species (proteins, complexes, RNA, small molecules), connected by edges representing activation or inhibition. A collection of paths will be extracted from the network, defined between annotated and target nodes of biological interest.
Experimental data	Experimental data related to processes described in the network. Species in the network can be annotated with experimental data values (consisting of a real number), such as an expression ratio or a *t*-test statistic value.

Input data types description for the calculation of the PIQuant score.

$$\text{PIQuan}t_{\text{Score}}=\overset{q}{\underset{k=1}{\sum }}\alpha_{k}\sigma_{k}\frac{1}{\lambda_{k}}$$

**Figure 5:**
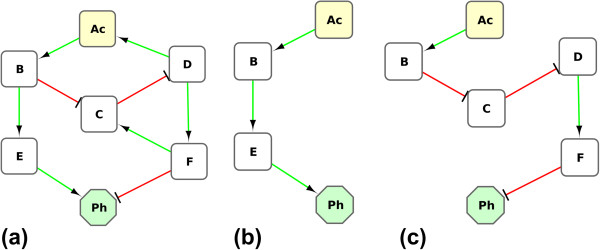
**A simple influence network.** The network is composed of seven nodes and nine edges **(a)**. The two paths **(b,c)** extracted from this network start from the annotated node **Ac** and end at the target node **Ph** (which denotes a phenotype of interest). The node **Ac** is annotated using experimental data, and is assigned the value 2.0.

In the case of the network presented in Figure 5a, let us consider Ac the annotated node and Ph the target node and consider only the two paths defined in the Figures 5b and 5c. Given that the node Ac is annotated by the value 2.0, that the first path has a length equal to 3, and that the second path has a length equal to 5, we can calculate the PIQuant score of the node Ac to the node Ph as:

$$\text{PIQuan}t_{\text{Score}}=2\cdot 1\cdot \frac{1}{3}+2\cdot (-1)\cdot \frac{1}{5}=0.27$$

In more realistic situations, we would have multiple annotated nodes with different annotations, and also multiple target nodes representing phenotypes of particular nodes of interest. We have implemented in BiNoM a set of functions that allow users to select annotated nodes, select target nodes, and choose among three different options for searching paths (shortest paths, optimal and suboptimal shortest paths, all non self-intersecting paths). The software is then calculating PIQuant scores for every target node specified, taking into account every possible path found by the algorithm. An interactive window is detailing the PIQuant score results, both globally and for every path from the annotated nodes to the target nodes. It is also possible to get a full text report detailing all the calculations and the results. We describe a detailed and concrete application of the PIQuant algorithm to a real biological network in the Results section.

### BiNoM BioPAX utils & query

The BioPAX format was primarily conceived as a standard facilitating the exchange of data between various database systems [8]. As a consequence, this format was designed first to be machine-readable, but was not intended to be edited and modified by biologists. Furthermore, due to its adoption by large biological knowledge repositories, some BioPAX files can be really large, such as the *Homo sapiens* network from the Reactome database [4] that has more than 6,000 reactions involving more than 8,000 chemical species (proteins, RNA molecules, metabolites, etc.).

**Figure 6:**
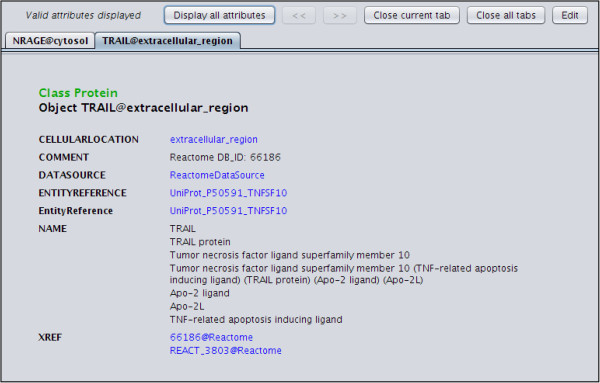
**Extra information linked to the TRAIL protein.** The information is automatically extracted from the BioPAX file upon import with the BiNoM I/O functions.

BiNoM implements a set of functions precisely aiming at allowing end users to easily visualize and modify BioPAX files. The functions are using Java class introspection techniques to build a BioPAX class tree. Then, the content of the file can easily be accessed. For example, Figure 6 shows all the information linked to the TRAIL protein, after a call to the BioPAX property editor function of the BiNoM menu has been made (for more details see the BiNoM manual available at (http://binom.curie.fr).

**Figure 7:**
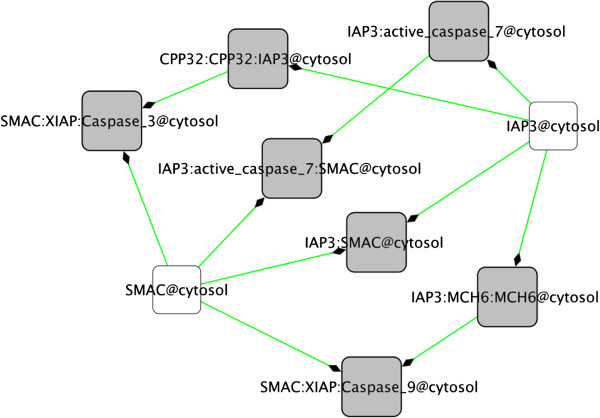
**SMAC pathway subnetwork.** Subnetwork extracted from the human apoptosis pathway, starting with the SMAC protein (white square) and expanding to all protein complexes where this molecule is involved (grey squares) using the BiNoM query functions.

The BioPAX query functions in BiNoM allow the user to work with huge BioPAX data files and extract the relevant information, by querying an index and retrieving data from it. The index corresponds to a mapping of the content of the BioPAX file on a labeled graph (an index file is created and saved, using the XGMML format). Various statistics can be displayed on the content of the index, such as the number of proteins, complexes, reactions, publications, etc. To start extracting relevant information, the user can query the index by gene name (and/or by any synonym of the gene) and start building a network centered around this molecule of interest. The extension of the network is done by adding different types of entities: complexes where the molecule of interest is involved, chemical species, reactions (with the possibility of including all the sources and targets of the reactions) and related publications. Figure 7 illustrates an example of a small network extracted from the human apoptosis pathway downloaded from the Reactome database [4], and centered on the SMAC protein, with all the protein complexes in which this protein is involved and that were added using the BiNoM BioPAX query functions.

### BiNoM module manager

To facilitate the visualization of large molecular networks, we propose a set of functions that simplify them by creating modules from selected parts of the large network. This task, that we call *modularization*, is a semi-automatic procedure, where biological expert knowledge is used to insure the coherence of the newly created modules.

Most of the modules represent a detailed sequence of events that occur with a particular protein or protein complex, whose name can then be used to represent the whole module (although any name can be used). This way, a simplified representation of a complex map can be produced, using the modules and their relationships as an abstracted version of the comprehensive network [18].

To facilitate the creation and management of modules, we have used in this version of BiNoM a new feature introduced in recent versions of Cytoscape (as of version 2.7) [22], known as *nested* networks. This feature allows the user to embed any cytoscape network in a (meta)node. It was introduced for the creation of network hierarchies and circular relationships. In BiNoM, we use this feature to facilitate the process of modularization of a large network. The BiNoM module manager integrates functions that allow users to easily create a network from a list of subnetworks, packing individual nodes, merging different subnetworks, displaying information about metanodes and calculating the intersection between subnetworks.

### BiNoM utilities

This set of functions corresponds to various small utilities that are not implemented in Cytoscape yet, but might be very useful for the analysis and manipulation of networks. For example, it is possible to automatically select all the edges between two nodes in the network in one operation, to generate the network corresponding to the double network differences between two networks A and B (creation of the two networks corresponding to *A*−*A*∩*B* and *B*−*A*∩*B*), or update all the subnetworks of a session after some changes have been made to the initial one. The BiNoM Utilities also implement clipboard functions, giving the possibility to copy, add and paste selected nodes and edges and also to show the clipboard content.

## Results and discussion

As an example of the use of BiNoM functions, we propose to study a reaction network focusing on the transition from G1 phase (growth phase) to S phase (DNA replication phase) of the cell cycle [18].

In a previous work, we have published a comprehensive map of the regulation of the well-known and charaterized tumor suppressor gene retinoblastoma (RB or RB1) [18]. The product of this gene operates at the heart of the cell cycle, acting as a signal transducer, connecting the cell cycle with the transcriptional machinery. The pathway in which RB is acting is disrupted in many human tumor types [38]. We used the Celldesigner software to create the map [20]. It lists 80 proteins, 208 chemical species, 165 interactions, 176 genes, and recapitulates more than 350 publications, including information from different cellular types, thus making the map a generic map of the cell cycle regulation. It is composed of two main compartments: the cell, containing the cytoplasm, the nucleus and the nucleolus, in which the biochemical interactions such as association, dissociation, (de)phosphorylation, (de)acetylation, degradation, etc. take place; and the genes, which lists the target genes of the main transcription factors of the map, the E2F family members. A thorough description of the model, the methods used to build it and create simplified versions of it along with an interactive (clickable) map are available on our websites (http://bioinfo-out.curie.fr/projects/rbpathway/) and http://navicell.curie.fr/navicell/maps/rbe2f/master/).

**Figure 8:**
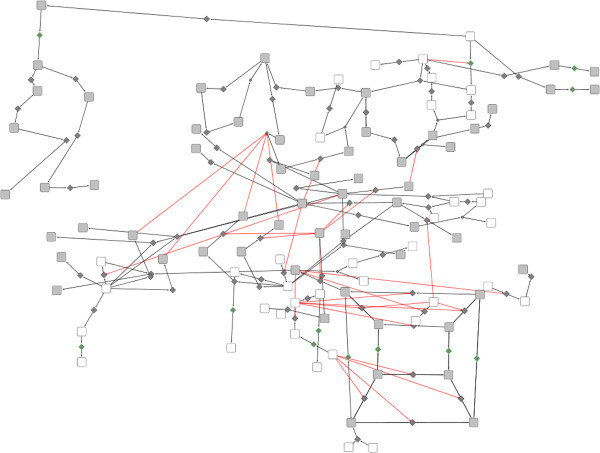
**G1/S network.** Overview of the G1 to S transition network, corresponding to the intersection of RB/E2F network and G1/S network extracted from Reactome.

For the study presented here, we chose to concentrate on the G1 to S transition. We used the intersection of the 208 chemical species of RB/E2F network and the 280 chemical species listed in Reactome [4] for the G1-S transition (referred to as *Mitotic G1 G1/S phases* in Reactome). The resulting subnetwork contains 38 proteins, 98 chemical species, and 100 biochemical reactions (Figure 8).

This map contains a lot of valuable information but it can be rather difficult to extract. We present two ways to get some biological insight from this map, by using BiNoM functions. The first one consists of transforming the reaction network into an influence network in order to analyze experimental data on it. The second one presents a simplification of the comprehensive map by applying a method of reduction of the numerous interactions into modules without losing any content from the original map.

### Application of PIQuant on an influence network

PIQuant algorithm can be used to perform a quantitative analysis on an influence network. In order to translate the G1/S reaction network into an influence network, we used a tool developed by the team of BIOCHAM [39] that is available online (http://contraintes.inria.fr/âˆ¼soliman/cd2dot.html). To translate a reaction network into an influence network, the former is pre-processed according to simple rules: (1) BIOCHAM deletes all non-regulated degradation and syntheses reactions, (2) all intermediary chemical species with only one input and one output are suppressed, (3) if the reactions of synthesis and degradation of the chemical species deleted in (2) have distinct inputs and outputs, then these reactions can be merged, and (4) if they have the same chemical species as input/output, then the reaction is a reversible reaction and is replaced by a degradation [40]. A thorough description of the procedure together with an example of such a conversion is available in [41].

**Figure 9:**
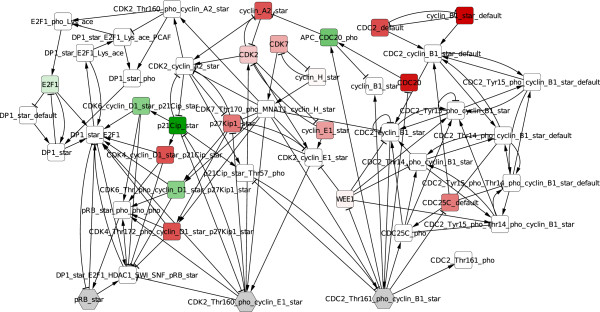
**Annotated influence network.** Influence network of the cell cycle G1/S transition generated by BIOCHAM. Colors represent differential expression obtained from transcriptomic data compared to normal tissue. Color intensities are proportional to the *t*-test statistic values (red values indicate positive values corresponding to an activation, green values indicate negative values corresponding to an inhibition). The three grey nodes are markers for the different cell cycle phases: G1 (pRB_star), S (CDK2/Cyclin E1 complex phosphorylated), M (CDC2/Cyclin E1 complex phosphorylated.

We applied PIQuant to the resulting influence network of the G1/S transition of the cell cycle (Figure 9). We selected three target nodes as markers of the G1, S and M phases of the cell cycle. For the experimental data, we used expression data from a study of 57 bladder cancer tissue samples compared to 4 normal samples [42]. For each gene, the differential expression between tumor and normal tissue is assessed by a *t*-test. The *t*-test statistic value is used as the annotation for each node. We selected the 19 nodes for which we had experimental data values as annotated nodes. Then, we constructed a text file listing nodes of the influence network and their annotation and we imported this file using the Cytoscape function “Import > Nodes attributes” in the Cytoscape session of the influence network. Figure 9 represents this influence network after its import.

**Figure 10:**
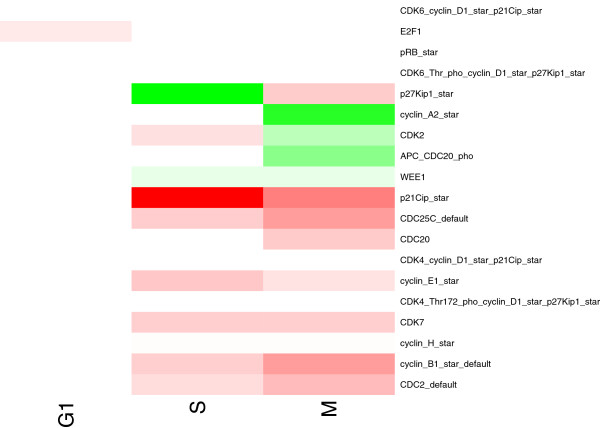
**Heatmap representation of the PIQuant scores.** The map shows the results of the PIQuant algorithm applied to the G1/S influence network. Color intensities are proportional to PIQuant score (red color indicates a positive value; i.e. an activation, the green color indicates a negative value, i.e. an inhibition). Each line represents an annotated node while each column represents a cell-cycle phase phenotype: G1 (pRB_star), S (CDK2/Cyclin E1 complex phosphorylated), M (CDC2/Cyclin E1 complex phosphorylated).

PIQuant is applied to this network and its annotation, by using the function “Plugins > BiNoM 2.1 > BiNoM Analysis > Path Influence Quantification analysis”. We selected the option “optimal and suboptimal shortest path” as the algorithm to extract the paths. The PIQuant score is then automatically calculated for each association between an annotated node and a target node. The user can browse the results on an interactive window detailing the different paths and their scores, and can also get a complete report, detailing the global and individual PIQuant scores from each annotated node to each cell-cycle phase marker (for more details on the interactive window and the report, see the BiNoM manual). The global PIQuant score from each annotated source node to each target is represented as a heatmap on Figure 10 (the list of nodes and all the PIQuant score values corresponding to the heatmap Figure are available as Additional file 1: Table S1). We can see on this Figure that most genes have positive scores (red coloring on the heatmap), in cancer cells compared to normal cells, indicating that they influence positively the M and S phases, corresponding to an enhanced proliferation for tumor cells. A clear difference can be observed when comparing the heatmap to Figure 9, where the color values represent only experimental data values. The heatmap represents this time the integration of both experimental data and the network architecture through the PIQuant score.

### Modularization of the G1/S molecular map

The initial G1/S network is very detailed and may be hard to grasp at a first glance. To facilitate the analysis of the content, we propose to organize the reaction network as a modular network. The chemical species are clustered in groups, referred to as modules, in an semi-automatic manner, using BiNoM functions and biological knowledge. Each module represents in fact a sequence of events occuring with a particular protein. The modules are then linked by activating or inhibiting influences according to the information contained in the original diagram or derived from previous biological knowledge. A detailed tutorial on the construction of this modular network using BiNoM is described in the Additional file 2: Supplementary methods.

**Figure 11:**
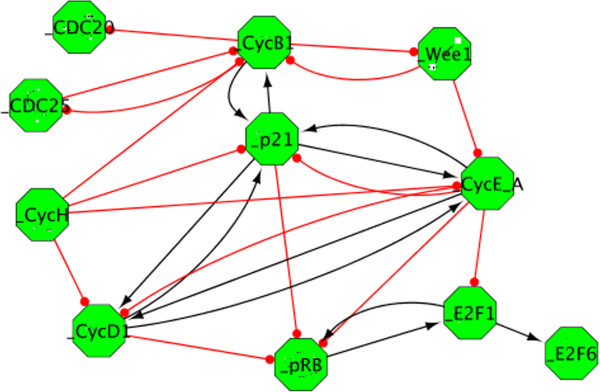
**Modular view of the G1/S network.** Modular representation of the G1/S network, created using a set of different BiNoM functions. Each node (pictured as a green octagon), represents a different module, or subnetwork. The edges connecting the modules represent the known influences between modules.

Briefly, we first decomposed the global network into its different components, by using name semantics, to isolate the subnetworks in which each protein is involved (decomposition in material components). The 33 networks that are created this way may share a lot of common chemical species, so we went further by clustering the subnetworks having at least 25% of common chemical species. We renamed the 9 clusters obtained with a name that illustrates the content and the main function of the clusters (such as E2F1_RB, Wee1, etc.). Then, we checked the content of each module, making modifications if necessary by adding or deleting nodes, according to specific biological knowledge. For example, the module E2F1_RB is further decomposed in three different modules containing the proteins RB, E2F1, and E2F6. Finally, we generated a modular view of all the individual modules, using the Module Manager functions. BiNoM links the modules if they share components or edges. These edges are then interpreted as activation or inhibition by the modeler. Our final modular view is composed of 11 modules, with 27 edges connecting them (Figure 11).

The modular view offers a simplified visualization of the complex network, without losing any information of the global map. The model obtained is more abstract but highlights some aspects that may not be evident from the comprehensive reaction network. For instance, it brings into relief feedbacks (positive, negative, or feedforward) involving the major players of the cell cycle, and prepares the network for mathematical modeling. The translation of this modular network into a Boolean model, for instance, is indeed straightforward. Another application for the modular model would be to analyze experimental data such as transcriptome or copy number variations (CGH). The “activity” of each module is based on the expression levels of the genes within the module, which can be visualized using a color code on the modular map. It’s then fairly easy to analyze the difference between a disease and a normal state, or even to try to discriminate between different disease stages. We have produced such maps for the RB/E2F modular network to analyze bladder tumor samples, and we could observe a striking difference between the non-invasive and invasive states of the disease [18] (the map and the details about the procedure can be seen at (http://bioinfo-out.curie.fr/projects/rbpathway/case_study.html).

## Conclusions

Building a useful model for systems and mathematical biology is a multi-step process, beginning with the collection of biological knowledge and progressing towards the formalization of a network and its translation in mathematical terms. BiNoM is designed to help during the intermediate steps of this process, by providing a convenient access to a selection of standard systems biology formats, by giving the possibility to analyze the network using various graph theory algorithms and map biological data onto it. BiNoM is clearly not a tool for numerical simulations, but it provides functions to export final networks to the SBML and GINsim file formats (through the GINsim Cytoscape plugin for Boolean modeling), facilitating the import into various numerical simulators. Together with functions described in this manuscript, BiNoM implements several other methods which are described elsewhere such as finding optimal minimal cut sets (http://bioinfo-out.curie.fr/projects/ocsana/), coloring CellDesigner maps, creating Google Maps-based interface for browsing large network maps (http://navicell.curie.fr) and finding enriched subnetworks [43].

## Availability and requirements

• Project name: BiNoM

• Project home page: (http://binom.curie.fr/)

• Operating system(s): Platform independent

• Programming language: Java

• Other requirements: Java 1.5 or higher, Cytoscape 2.7, 2.8

• License: GNU LGPL

• Any restriction to use by non-academics: none

## Competing interests

The authors declare that they have no conflict of interest.

## Authors’ contributions

EB, AZ and LC designed the study. EB, AZ and DR wrote the code. EB, AZ, LC, DR and GS generated the data, performed the analyses and interpreted the results. EB, AZ and LC wrote the manuscript. AZ and EmB supervised the study. All authors edited and approved the final version of the manuscript.

## Supplementary Material

Additional file 1. Table S1 PIQuant score values for all the annotated nodes (rows) and all the target nodes (columns) of the G1/S influence network. Click here for file

Additional file 2 **Supplementary methods.** Installation procedure for BiNoM, changelog for BiNoM version 2.0 compared to version 1.0 and detailed tutorial for the creation of a modular view of the G1/S network using BiNoM functions. Click here for file
