# Supplementary material for: BiNoM 2.0, a Cytoscape plugin for accessing and analyzing pathways using standard systems biology formats
Source: BMC Syst Biol. 2013 Mar 1;7:18. doi: 10.1186/1752-0509-7-18 (PMC3646686; doi:10.1186/1752-0509-7-18)
Supplement: Additional file 2 — Supplementary methods. Installation procedure for BiNoM, changelog for BiNoM version 2.0 compared to version 1.0 and detailed tutorial for the creation of a modular view of the G1/S network using BiNoM functions. [file 1752-0509-7-18-S2.pdf]

Supplementary methods for the manuscript "BiNoM, a Cytoscape plugin for accessing and analyzing pathways using standard systems biology formats"

December 21, 2012

Installation

BiNoM plugin can be installed in two different ways.

- Launch Cytoscape and then launch the plugin manager (menu "Plugins > Manage Plugins"). Choose the category "Other" in the left panel of the manager, and select the latest version of BiNoM. Click "Install" and restart Cytsocape.
- Download the BiNoM "jar" file from our website `http://binom.curie.fr` and copy it in the "plugins" directory of the Cytoscape installation folder. Restart Cytoscape.

Changelog

We provide here a list of the main changes that were introduced for the version of BiNoM described in this manuscript, as compared to the original version by Zinovyev et al. [1].

- Support for the export and import of BioPAX Level 3 files. Please note that versions 2.x of BiNoM accepts only BioPAX Level 3 files. The previous version of BiNoM (version 1.0) is available from our website (`http://binom.curie.fr`) for users that would like to process BioPAX Level 2 files.
- Module manager: a complete set of functions that simplify the visualization and modularization of large molecular networks. The functions rely on a new feature introduced with Cytoscape 2.7 and higher and knwon as *nested* networks.
- Path Influence Quantification algorithm (PIQuant): this algorithm was available in beta version in the previous version of BiNoM, but we provide now a stable version with detailed explanations on the algorithm and a case study in the manuscript.

- A complete case study illustrating the principal functions of BiNoM applied to the analysis of a network representing the G1/S transition of the cell cycle.

- Numerous bugs in the code have been fixed since the version 1.0 of BiNoM.

Tutorial for the modularization of the G1/S network using BiNoM functions

In this tutorial, we describe the step-by-step procedure to extract a compact modular network from a large network representing the cell cycle G1/S transition. The input file can be downloaded from our website `http://binom.curie.fr/`.

1. Import network.
   File → Import → Network (Multiple File Types)...
   Choose the file g1s_209_266.xgmml. The network "G1S" is created in Cytoscape.

2. Decompose the network into subnetworks.
   We choose to decompose the network according to the different components present in the map rather than decomposing according to the cycles of the network. **Plugins → BiNoM 2.1 → BiNoM Analysis → Get Material Components**.
   33 subnetworks are created.

3. Clustering.
   The resulting subnetworks may share a lot of species. To reduce the number of subnetworks, we cluster them with an overlap of 25%.
   Plugins → BiNoM 2.1 → BiNoM Analysis → Cluster networks.
   A pop-up window appears. Among the proposed networks, select all networks except G1S.xml and choose 25% overlap. 9 networks are created.

4. Rename the newly-created clusters. It is important to rename right away the clusters with a name that illustrates the content of each of these clusters. Choosing the appropriate name might be facilitated by listing the name of the components of the cluster: **Plugins → BiNoM 2.1 → BiNoM module manager → List components of species in network and modules**
   To rename them, right-click on the Network panel. We propose to use the following names for these 9 clusters: **E2F1_RB**, **CycD1**, **CycE_A**, **Wee1**, **p21**, **CycH**, **CycB1**, **CDC25** and **CDC20**.

5. Inside the clusters, check the content of the modules (manual curation of the modularization).

   - For cluster E2F1_RB, separate RB from E2F1 and from E2F6 and create three different modules. **Select → Nodes → By Name**. Write "*E2F6*". The nodes with E2F6 are highlighted.Select the reactions between the nodes. **Select → Nodes → First neighbours of selected nodes**. **File → New → Network → From selected**

nodes all edges. Rename "E2F1_RB child" to "E2F6" and delete the nodes from E2F1_RB network.

- Do the same for pRB.
- Rename the remaining network of E2F1_RB, E2F1.
- The resulting list of modules is: CDC25, CDC20, CycB1, Wee1, CycH, p21, CycD1, CycE_A, E2F1, RB, E2F6.

6. Select modules to prepare the modular view. In the pop-up window, choose the 11 modules. **Plugins → BiNoM 2.1 → BiNoM Module Manager→ Create Network of Modules**.

7. Connect modules. **Plugins → BiNoM 2.1 → BiNoM Module Manager → Create Connections between Modules.**

References

[1] Zinovyev A, Viara E, Calzone L, Barillot E: **BiNoM: a Cytoscape plugin for manipulating and analyzing biological networks**. *Bioinformatics* 2008, **24**(6):876.
